# Microglial ADGRG1/GPR56 regulates amyloid clearance in Alzheimer’s disease

**DOI:** 10.1002/alz.084250

**Published:** 2025-01-03

**Authors:** Beika Zhu, Andi Wangzhou, Diankun Yu, Tao Li, Rachael Schmidt, Stacy L. De Florencio, Lauren Chao, William W. Seeley, Xianhua Piao

**Affiliations:** ^1^ Eli and Edythe Broad Center of Regeneration Medicine and Stem Cell Research, University of California, San Francisco, San Francisco, CA USA; ^2^ Weill Institute for Neuroscience, University of California, San Francisco, San Francisco, CA USA; ^3^ Memory and Aging Center, UCSF Weill Institute for Neurosciences, University of California San Francisco, San Francisco, CA USA; ^4^ Department of Pathology, University of California, San Francisco, San Francisco, CA USA; ^5^ Newborn Brain Research Institute, University of California, San Francisco, San Francisco, CA USA; ^6^ Department of Pediatrics, University of California, San Francisco, San Francisco, CA USA

## Abstract

**Background:**

Alzheimer’s disease (AD) is a devastating neurodegenerative disease with virtually no therapeutic interventions to reverse its pathology. Emerging studies emphasize the role of glial cells, particularly microglia, in brain homeostasis and AD progression. Notably, the adhesion G protein‐coupled receptor GPR56 (also known as ADGRG1) is one of the critical genes defining yolk sac‐derived microglia. Recent single‐nucleus RNA sequencing (snRNAseq) studies have shown GPR56, as one of the top five genes upregulated in microglia in individuals with early‐stage AD compared to those with no pathology or late‐stage AD. This observation suggests that individuals with upregulated microglial GPR56‐mediated pathways may have survived to an advanced age with mild AD pathology. In the present study, we test the hypothesis that microglial GPR56 is critical in modulating the microglial response in AD.

**Method:**

We developed a novel AD mouse model with specific deletion of GPR56 in the microglia of 5xFAD mice (designated AD‐cKO and AD‐Control). Comprehensive analyses, including immunostaining, behavioral tests, and snRNAseq, were employed. The impact of GPR56 on microglial phagocytosis was evaluated using both *in vitro* and *in vivo* assays.

**Result:**

Absence of microglial GPR56 exacerbated AD pathology, characterized by a compromised microglial response, increased plaque burden, exacerbated neuronal pathology, and cognitive deficits. snRNAseq analysis revealed a downregulation of genes associated with microglial homeostasis, phagocytosis, and lysosomal functions in AD‐cKO mice. Phagocytosis assays showed a significant reduction in phagocytic capability in AD‐cKO microglia. Extending this assay to human iPSC‐derived microglia, we observed a corresponding phenotype in GPR56 KO cells. Pearson correlation coefficient analysis using several published human sequencing databases corroborated our results in mouse models.

**Conclusion:**

Our study supports that microglial GPR56 plays an indispensable role in curtailing AD progression, presenting a potential new therapeutic target in combating AD.

